# Supplementary material for: Subsampling scaling
Source: Nat Commun. 2017 May 4;8:15140. doi: 10.1038/ncomms15140 (PMC5418619; doi:10.1038/ncomms15140)
Supplement: Supplementary Information — Supplementary figures, supplementary notes and supplementary references. [file ncomms15140-s1.pdf]

Supplementary Note 1. Mathematical subsampling

In the following, we derive in detail the novel subsampling scaling. We first introduce the definition and basic results of subsampling in analogy to Stumpf et al. [1], who treated subsampling of graphs. We then extend the aforementioned study as follows:

- First, we focus on an analytical inference of the distribution of the full system from the subsampling, a topic that was not touched by Stumpf et al. To this end we derive (a) the exact subsampling scaling for negative binomials and exponentials, and (b) the approximate scaling for power-law distributions.
- Second, we explicitly show how to derive the system size from subsampling induced deviations from power laws ("hairs").
- Third, we treat the relation between subsampling and finite size effects.
- Last, we apply our analytically derived subsampling scaling ansatz to infer the probability distribution of avalanche sizes in developing neural networks.

Let X be a discrete, non-negative random variable with probability distribution $P(X = s) = P(s)$, with $s \in \mathbb{N}_0$, then $G(z) = \sum_{s=0}^{\infty} z^s P(s)$ is the corresponding probability generating function (PGF). For distributions such as power laws, where $s = 0$ is not supported, s is constrained to $s \in \mathbb{N}$, and the probability distribution needs to be normalized accordingly. X represents the size of a set of "events" that comprise a "cluster", e.g. the number of spikes in an avalanche, or the degree of a node. For subsampling, we assume that each of the events in the cluster is sampled independently with probability p, resulting in a random variable for the observed cluster size, X_{sub} [1]. Thus the probability $P_{\text{sub}}(X_{\text{sub}} = s)$ to observe a cluster of size s is derived using a binomial distribution:

$$P_{\text{sub}}(s) = \sum_{k=s}^{\infty} P(k) \binom{k}{s} p^s (1-p)^{k-s}.$$

The PGF $G_{\text{sub}}(z; p)$ for X_{sub} with given p is thus:

$$\begin{aligned}
G_{\text{sub}}(z;p) &= \sum_{s=0}^{\infty} z^s P_{\text{sub}}(s) \\
&= \sum_{s=0}^{\infty} z^s \sum_{k=s}^{\infty} P(k) \binom{k}{s} p^s (1-p)^{k-s} \\
&= \sum_{k=0}^{\infty} P(k) \sum_{s=0}^{k} z^s \binom{k}{s} p^s (1-p)^{k-s} \\
&= \sum_{k=0}^{\infty} P(k) (zp + (1-p))^k,
\end{aligned}$$

Thus the PGF of X and X_{sub} show a direct relation [1]:

$$G_{\text{sub}}(z;p) = G(1 - p(1-z)) \tag{1}$$

As a consequence, the expected values of X and X_{sub} also are closely related: Using the expression for the expected value of X, $E(X) = G'(1^-)$

$$E(X_{\text{sub}}) = G'_{\text{sub}}(1^-;p) = G'(1-p(1-1^-)) = pG'(1^-) = pE(X) \tag{2}$$

These relations hold for any $P(s)$, however, only for specific $P(s)$, namely positive and negative binomials, the full and subsampled system's $P(s)$ follow the same family of distributions [1], e.g. if $P(s)$ is a binomial distribution, then $P_{\text{sub}}(s)$ also is a binomial, but with different parameters.

Subsampling of negative binomial and exponential distributions

Assuming that X follows a negative binomial distribution $X \sim \text{NB}(r, p_{\text{NB}})$,

$$P(X = s) = \binom{s+r-1}{s} p_{\text{NB}}^r (1 - p_{\text{NB}})^s, \tag{3}$$

then the expectation of X is given by

$$m = E(X) = r\frac{(1-p_{\text{NB}})}{p_{\text{NB}}},$$

and the PGF is

$$G(z) = \left(\frac{p_{\text{NB}}z - z + 1}{p_{\text{NB}}}\right)^{-r} = \left(1 + \frac{m}{r}(1-z)\right)^{-r}.$$

Using Eq. 1 then returns the PGF under subsampling,

$$G_{\text{sub}}(z;p) = \left(1 + \frac{pm}{r}(1-z)\right)^{-r},$$

which corresponds to the negative binomial distribution with the same r, but different p_{NB}, selected such that

$$\frac{1-p'_{\text{NB}}}{p'_{\text{NB}}} = p\frac{1-p_{\text{NB}}}{p_{\text{NB}}}. \tag{4}$$

In the special case of $r = 1$, the negative binomial is a geometric distribution with probability parameter p_{NB}, and the discrete exponential distribution is a particular parametrization of the geometric distribution $1 - p_{\text{NB}} = e^{-\lambda}$.

$$P_{\exp}(s) = (1 - e^{-\lambda}) \cdot e^{-\lambda s}, \text{ with } s \in \mathbb{N}.$$

Using equation 4, the relation between λ and λ_{sub} is:

$$\frac{e^{-\lambda_{\text{sub}}}}{1-e^{-\lambda_{\text{sub}}}} = p\frac{e^{-\lambda}}{1-e^{-\lambda}} \Leftrightarrow e^{\lambda_{\text{sub}}} - 1 = \frac{e^{\lambda}-1}{p}.$$

Solving this equation with respect to λ_{sub} we obtain:

$$\lambda_{\text{sub}} = \ln\left(\frac{e^{\lambda}+p-1}{p}\right). \tag{5}$$

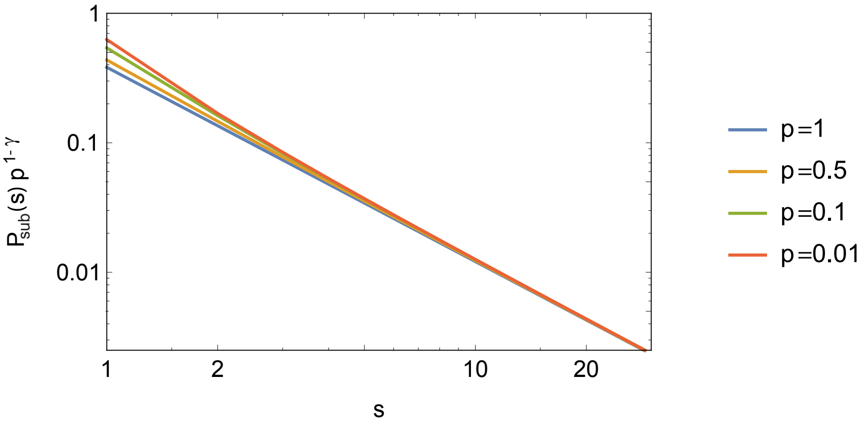

Supplementary Figure 1: Scaling of subsampled power-law distributions $P_{\text{sub}}(s)$, using $a = 1 - \gamma$, $b = 0$, with $\gamma = 1.5$. the blue line shows the perfect power law of the fully sampled distribution, i.e. $P(s)$. Note the deviations from power law for small s, which increase with smaller sampling probability p.

Subsampling of power-law distributions

To derive an approximate scaling for power-law distributions under subsampling, we expand on the work by Stumpf et al. [1]. Consider mathematical subsampling as defined in the main text, and a power-law distribution $P(s) = C_\gamma s^{-\gamma}$ with exponent $\gamma > 1$, and normalization $C_\gamma = 1/\zeta(\gamma)$, where $\zeta(\gamma)$ is the Riemann zeta function. Then $P_{\text{sub}}(s; \gamma, p)$ is a binomial subsampling with sampling probability p:

$$P_{\text{sub}}(s; \gamma, p) = C_\gamma \sum_{n=0}^{\infty} (s+n)^{-\gamma} p^s (1-p)^n \binom{s+n}{n}. \tag{6}$$

Building on the work by Stumpf et al., we assume that for the tail (i.e. for $s \to \infty$) the subsampled distribution is approaching an appropriately scaled power-law with slope $\gamma_{\text{sub}} = \gamma$, i.e. we assume

$$P_{\text{sub}}(s; \gamma, p) \stackrel{s\to\infty}{\longrightarrow} c_\gamma(p) s^{-\gamma}. \tag{7}$$

$c_\gamma(p)$ is the subsampling-dependent normalization constant. To derive how $c_\gamma(p)$ depends on p, we need to assume that

$$\frac{\partial}{\partial p} P_{\text{sub}}(s; \gamma, p) \stackrel{s\to\infty}{\longrightarrow} s^{-\gamma} \frac{\partial}{\partial p} c_\gamma(p). \tag{8}$$

This is a strong assumption, because typically an exchange of differentiation and limit is only possible in case of uniform convergence of the derivatives [2], which is not the case here. However, all the functions we consider are monotonous in all parameters and numerical results support the assumption above.

In the following we assume that s is large enough so that Eq. 7 can be taken as an identity. Then

$$\begin{aligned} \frac{\partial}{\partial p} P_{\text{sub}}(s; \gamma, p) &= C_\gamma s \sum_{n=0}^{\infty} (s+n)^{-\gamma} p^{s-1} (1-p)^n \binom{s+n}{n} \\ &- C_\gamma \sum_{n=0}^{\infty} (s+n)^{-\gamma} p^s n (1-p)^{n-1} \binom{s+n}{n}. \end{aligned}$$

The first term can be approximated as:

$$\begin{aligned} & C_\gamma s \sum_{n=0}^{\infty} (s+n)^{-\gamma} p^{s-1} (1-p)^n \binom{s+n}{n} \\ = \ & \frac{s}{p} P_{\text{sub}}(s;\gamma,p) \approx \frac{s}{p} c_\gamma(p) s^{-\gamma}. \end{aligned}$$

The second term, after introducing $k = n - 1$, reduces to:

$$\begin{aligned} & C_\gamma \sum_{n=0}^{\infty} (s+n)^{-\gamma} p^s n (1-p)^{n-1} \binom{s+n}{n} \\ = \ & C_\gamma \frac{s+1}{p} \sum_{k=0}^{\infty} (s+1+k)^{-\gamma} p^{s+1} (1-p)^k \binom{s+1+k}{k} \\ = \ & \frac{s+1}{p} P_{\text{sub}}(s+1;\gamma,p) \\ \approx \ & \frac{s+1}{p} c_\gamma(p)(s+1)^{-\gamma} \end{aligned}$$

The "≈" is inherited from Eq. 7, which is only exact for $s \to \infty$. Combining the two terms, we obtain for Eq. 8:

$$s^{-\gamma} \frac{\partial}{\partial p} c_\gamma(p) \approx \frac{\partial}{\partial p} P_{\text{sub}}(s;\gamma,p) \approx \frac{s}{p} c_\gamma(p) s^{-\gamma} - \frac{s+1}{p} c_\gamma(p)(s+1)^{-\gamma}. \tag{9}$$

From this, $\frac{\partial}{\partial p} c_\gamma(p)$ can be expressed as:

$$\frac{\partial}{\partial p} c_\gamma(p) = \frac{c_\gamma(p)}{p} \lim_{s\to\infty} \left[s - (s+1)\left(1 + \frac{1}{s}\right)^{-\gamma} \right] \tag{10}$$

For solving the limit, we use the known identity

$$\lim_{x\to 0} \frac{(1+x)^\mu - 1}{\mu x} = 1,$$

which can be restated by replacing x by $1/s$ and μ by $-\gamma$:

$$\lim_{s\to\infty} s\left(1 + \frac{1}{s}\right)^{-\gamma} - s = -\gamma.$$

Thus

$$\frac{\partial}{\partial p} c_\gamma(p) = \frac{c_\gamma(p)}{p}(\gamma - 1) \tag{11}$$

This differential equation is solved by:

$$c_\gamma(p) = C^* p^{\gamma-1}, \tag{12}$$

For $p = 1$ we know that $C^* = C_\gamma$, because sampling all units does not change the distribution. The final expression for $c_\gamma(p)$ is thus

$$c_\gamma(p) = C_\gamma p^{\gamma-1}. \tag{13}$$

With this we can derive scaling parameters a, b that collapse the distribution's tails, i.e. $p^a P_{\text{sub}}(p^b s) = P(s)$ for large s. Using Eq. 13:

$$p^a P_{\text{sub}}(p^b s) = C_\gamma p^{\gamma-1} p^a (p^b s)^{-\gamma} = p^{a-b\gamma+\gamma-1} C_\gamma s^{-\gamma}. \tag{14}$$

Thus for any a and b, such that $a - b\gamma = 1 - \gamma$, the scaling ansatz leads to a collapse. One of the members of this scaling family is $b = 0$, $a = 1 - \gamma$, which scales the y-axis only. As shown in Supplementary Fig. 1, this scaling collapses the tails of distributions perfectly. For small s, however, there are systematic deviations under subsampling, which increase with smaller p. We call them "hairs", because they grow on the head of the distribution, as opposed to the tails.

A different member of the scaling family is $a = b = 1$. This scaling is especially attractive, because it does not require information about the exponent γ of the power law (see Supplementary Fig. 1). As this scaling is linear in p, we call it p-scaling.

Power-law exponent close to unity

Here we show why the "hairs" become smaller, i.e. converge to zero, in the limit of the power-law exponent $\gamma \to 1$. It is in agreement with results of Stumpf et al. [1], stating that "hairs" are growing with increase of the exponent. Mathematically, the exponent of the power-law distribution cannot be exactly equal to one or smaller, because in this case the distributions cannot be normalized. Thus without loss of generality we consider truncated power laws: $P(s) = C \cdot s^{-1}$ for $s \leq s_{\max}$ and $P(s) = 0$ for $s > s_{\max}$. The normalizing constant C depends on $s_{\max}$. In this case the subsampled distribution $P_{\text{sub}}(s)$, with sampling probability p can be written explicitly

$$P_{\text{sub}}(s; p) = \sum_{l=s}^{s_{\max}} \frac{C}{l} \binom{l}{s} p^s (1-p)^{l-s} = \frac{C}{s} \sum_{l=s}^{s_{\max}} \binom{l-1}{s-1} p^s (1-p)^{l-s}.$$

We are interested in the behavior of the "hairs" and thus consider small s. In this case, we can approximate $P_{\text{sub}}(s; p)$ by the infinite sum, and make use of the geometric series

$$\frac{1}{(1-x)^s} = \sum_{n=0}^{\infty} \binom{n+s-1}{s-1} x^n$$

to obtain, with a variable exchange $m = l - s$,

$$P_{\text{sub}}(s; p) \approx \frac{C}{s} p^s \sum_{m=0}^{\infty} \binom{m+s-1}{s-1} (1-p)^m = \frac{C}{s} = P(s).$$

Thus we showed that in the limit $\gamma \to 1$ subsampling of the power law converges to the original power law.

Inferring the system size from the subsampled distribution

The deviations from power laws (i.e. the hairs), which emerge under subsampling, allow to infer the system size M from the subsampled distribution $P_{\text{sub}}(s)$ alone, given that $P(s)$ follows a power law. This is because the hairs are a function of

the sampling probability $p = N/M$. The hairs are most pronounced for $P_{\text{sub}}(s=1)$ (except for $P_{\text{sub}}(s=0)$, which may remain unknown in experiments). Therefore, the inference of system size in experiments is most accurate if it is based on $P_{\text{sub}}(s=1)$. We explore this in the following derivations. Derivations based on other (small) s can be performed analogously.
Quantitatively, using the explicit relation for subsampling of power laws (Eq. 6) with $l = n+1$ results in:

$$\begin{aligned} P_{\text{sub}}(s=1) &= \sum_{l=1}^{\infty} \frac{l^{-\gamma}}{\zeta(\gamma)} l (1-p)^{l-1} p \\ &= \frac{p}{(1-p)\zeta(\gamma)} \sum_{l=1}^{\infty} \frac{(1-p)^l}{l^{\gamma-1}} \\ &= \frac{p \cdot Li_{\gamma-1}(1-p)}{(1-p)\zeta(\gamma)}, \end{aligned}$$

where ζ is the Riemann zeta function, and $Li_\gamma(z) = \sum_{k=1}^{\infty} z^k/k^\gamma$ is the polylogarithm function. This relation is exact if $P(s)$ is a true power law. For application to the real data obtained from subsampled observation the following algorithm allows to infer p:

1. Check whether the experimentally obtained empirical distribution $P_{\text{emp}}(s)$ is likely to originate from a system that under full sampling shows a power-law distribution. If not, the method cannot be applied.

2. Estimate the power-law slope γ of the power-law tail of the distribution to obtain $\hat{\gamma}$.

3. Solve the following equation for p:

$$P_{\text{emp}}(s=1) = \frac{p \cdot Li_{\hat{\gamma}-1}(1-p)}{(1-p)\zeta(\hat{\gamma})}$$

This will return the sampling probability p. From this, the system size can be inferred if N is known. This approach is also applicable approximately if the full system does not display a pure power law, but a power law with cutoff at large s. Then the power-law slope γ has to be inferred on an appropriate interval between the hairs and the cutoff.
We applied this method numerically to the data generated by the critical branching model of size $M = 1024$, subsampled to $N = 2^0, 2^1, \ldots 2^9$ units based on 10^7 avalanches in the full system. Indeed, the full system size could be inferred by $\hat{M} = \hat{p}N$ with high precision (Fig 2): The maximal deviations were smaller than 6%.

Supplementary Note 2. Subcritical systems

As outlined in the main text, avalanche distributions collapse under p-scaling for critical systems, but not for subcritical systems. The main reason is that for subcritical systems the exponential tail is too steep, i.e. the requirement $\lambda \ll p$ is

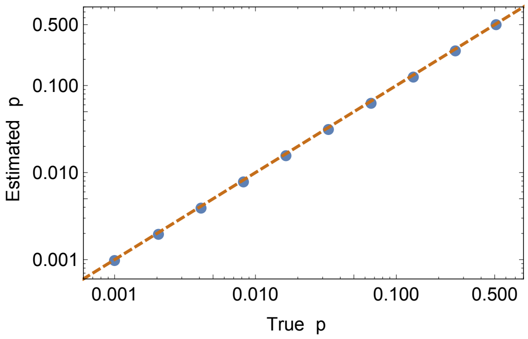

Supplementary Figure 2: Estimating the sampling fraction p from the "hairs". Dots represent the estimated p as a function of the true one, the dashed line represent the perfect correspondence.

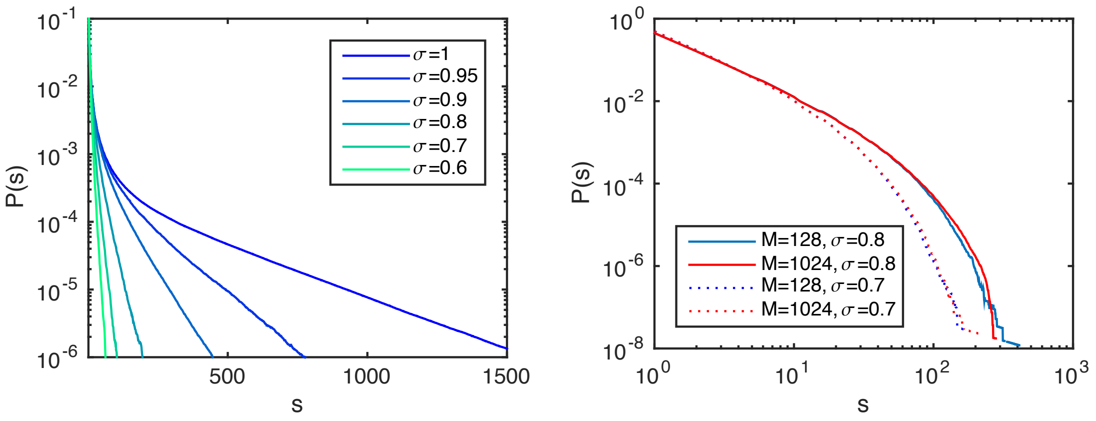

Supplementary Figure 3: Exponential tails of subcritical distributions. Left: Subcritical distributions for different branching ratios σ plotted in a log-lin scale clearly show exponential tails, with the tail slope λ depending on σ (results for $M = 1024$). Right: Subcritical distribution with a fixed deviation from criticality ($\sigma = 0.7$ or 0.8) for different system sizes M.

violated. We in the following derive an approximate relation between λ and the distance to criticality ($\varepsilon = \sigma_{\text{crit}} - \sigma = 1 - \sigma$). We show that for more subcritical systems, λ becomes increasingly larger (see also Supplementary Fig. 3 (left)). To approximately derive the relation between λ and ε (or σ), we used the branching process [3], because it allows to easily control the distance to criticality by changing the branching ratio σ, and because it is independent of finite size effects. This is a reasonable assumption, because in subcritical systems $P(s)$ is not affected by changing the system size for any $M > M_0$. Only for very small systems sizes there are finite size effects (Supplementary Fig. 3 (right)).

To derive heuristically the slope of the exponential tail λ as a function of the control parameter σ, consider a branching process with branching ratio $\sigma < 1$, and assume that an avalanche starts with 1 neuron firing. Then on expectation in the second time step there are σ neurons firing, in the third time step σ^2, and so forth. Thus we obtain an expression for the average avalanche size $\langle s \rangle$:

$$\langle s \rangle = 1 + \sigma + \sigma^2 + \sigma^3 + \ldots = \frac{1}{1 - \sigma}.$$

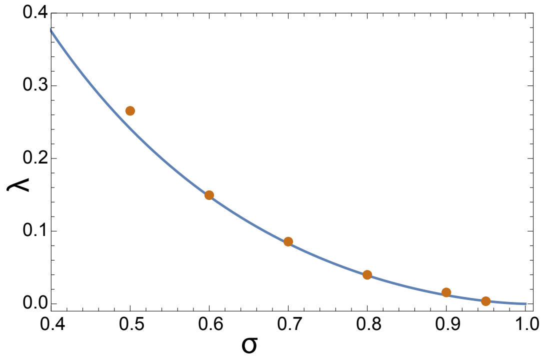

Supplementary Figure 4: Slope of tails, λ, for avalanche distributions of subcritical models. λ increases with increasing distance to criticality (decreasing σ). The dots denote the numerical results for 10^7 avalanches on the full system, the line denotes the analytical results.

In the subcritical regime, the distribution of the avalanche sizes is dominated by the exponential cutoff. We consider that they are well approximated by the power law with slope γ and an exponential cutoff parametrized by λ

$$P(s) = C_{\text{norm}} s^{-\gamma} e^{-\lambda s}.$$

The mean value of this distribution is given by:

$$\langle s \rangle = \frac{Li_{1-\gamma}(e^{-\lambda})}{Li_{\gamma}(e^{-\lambda})}$$

Where $Li_{\gamma}(z) = \sum_{k=1}^{\infty} z^k/k^{\gamma}$ is again the polylogarithm function. The relation between λ and σ is thus

$$\frac{Li_{1-\gamma}(e^{-\lambda})}{Li_{\gamma}(e^{-\lambda})} = \frac{1}{1-\sigma}, \tag{15}$$

and hence λ approaches zero when approaching the critical point ($\sigma \to 1$). As λ decays slowly as a function of σ, except in the very close vicinity of the critical point, the requirement for p-scaling, $\lambda \ll p$, is only satisfied in the close vicinity of the critical point. Else p-scaling does not apply.
To compare our analytical with numerical results, we used the same data as in Supplementary Fig. 3. We first estimated $\gamma \approx 1.3$ from the distributions, and with this solved equation 15 numerically. The analytical results closely fitted the slopes λ of the exponentials from the simulations (Supplementary Fig 4).

Supplementary Note 3. Subsampling of the EHE-model and sparse branching model

In this section, we investigate whether subsampling scaling also applies to other models than the ones treated in the main manuscript. In particular, we treat here first the Eurich, Herrmann & Ernst (EHE) model [4], a classical extension of the BTW model to neural networks, and then a realization of the BM with sparse connectivity ($k = 4$, see Methods). The details of the EHE model can be found in [4,

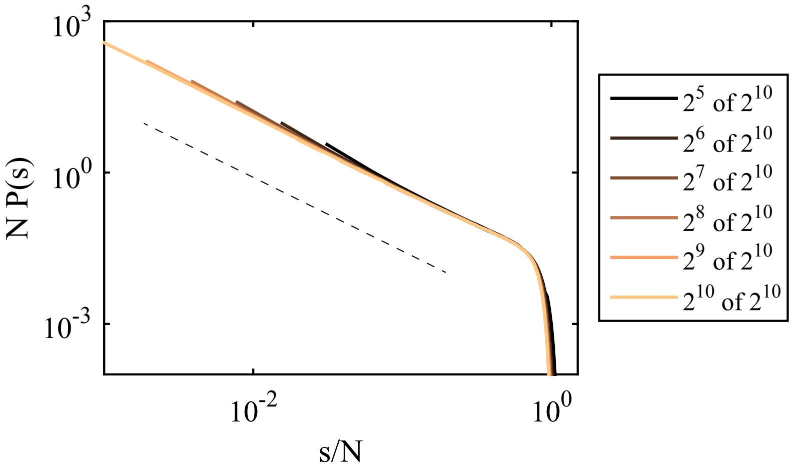

Supplementary Figure 5: Subsampling scaling of the Eurich, Herrmann & Ernst model. $N = 32, 64, \ldots, 512$ units were sampled out of $M = 1024$ units. Parameters: connection probability $p_{\text{conn}} = 0.1$, connection strength $\alpha = 0.96/(M \cdot p_{\text{conn}})$. The dashed line indicates a slope of -1.5.

5]. This model produces power-law distributions of the avalanche sizes with slope ≈ 1.5 that indicates that it belongs to the same university class as the branching model. However, activity transmission is not stochastic as in BM, but deterministic as in BTW. Another peculiarity of the model lays in its dissipative nature: for the finite system sizes M each spike leads to dissipation of $\Delta \approx 1/\sqrt{M}$ [4]. Thus only in the limit $M \to \infty$ the model is both truly critical and conservative. We simulated the EHE model with both, the classical fully connected graph topology and also with random connectivity probability p_{conn}. For both models, the dynamics is as follows: Each neuron i is a non-leaky integrator, and its membrane potential is denoted by $h_i \in [0, 1)$. When h_i crosses the threshold $\theta = 1$ the neuron fires and is reset $h_i \mapsto h_i - 1$ and all its postsynaptic connections receive an input of strength α/M, where α is the control parameter in the model, the strength of interaction. For the fully connected network, it is known that $\alpha \approx 1 - M^{-0.5}$ leads to an approximate power-law distribution of the avalanche sizes. For not-fully connected networks the connection probability p_{conn} needs to be included, and thus the condition to achieve approximate power-law distributions generalizes to $\alpha \cdot p_{\text{conn}} \approx 1 - M^{-0.5}$.

As the avalanche size distribution in the EHE model can be directly mapped to the branching model [5], subsampling scaling is expected to behave the same as in the BM, producing "hairs" but resulting in a good collapse. We tested this for the model of $M = 1024$ neurons with $p_{\text{conn}} = 0.1$ and obtained, as expected, a collapse under subsampling scaling (Supplementary Fig. 5).

The distributions of the fully and the sparsely connected BM are very similar (Supplementary Fig. 6). The only difference is a slightly more pronounced lack of small avalanches in the fully sampled sparse BM (Supplementary Fig. 6 C), which translates to somewhat less pronounced "hairs", in particular under "mild" subsampling ($N \geq 2^{10}$).

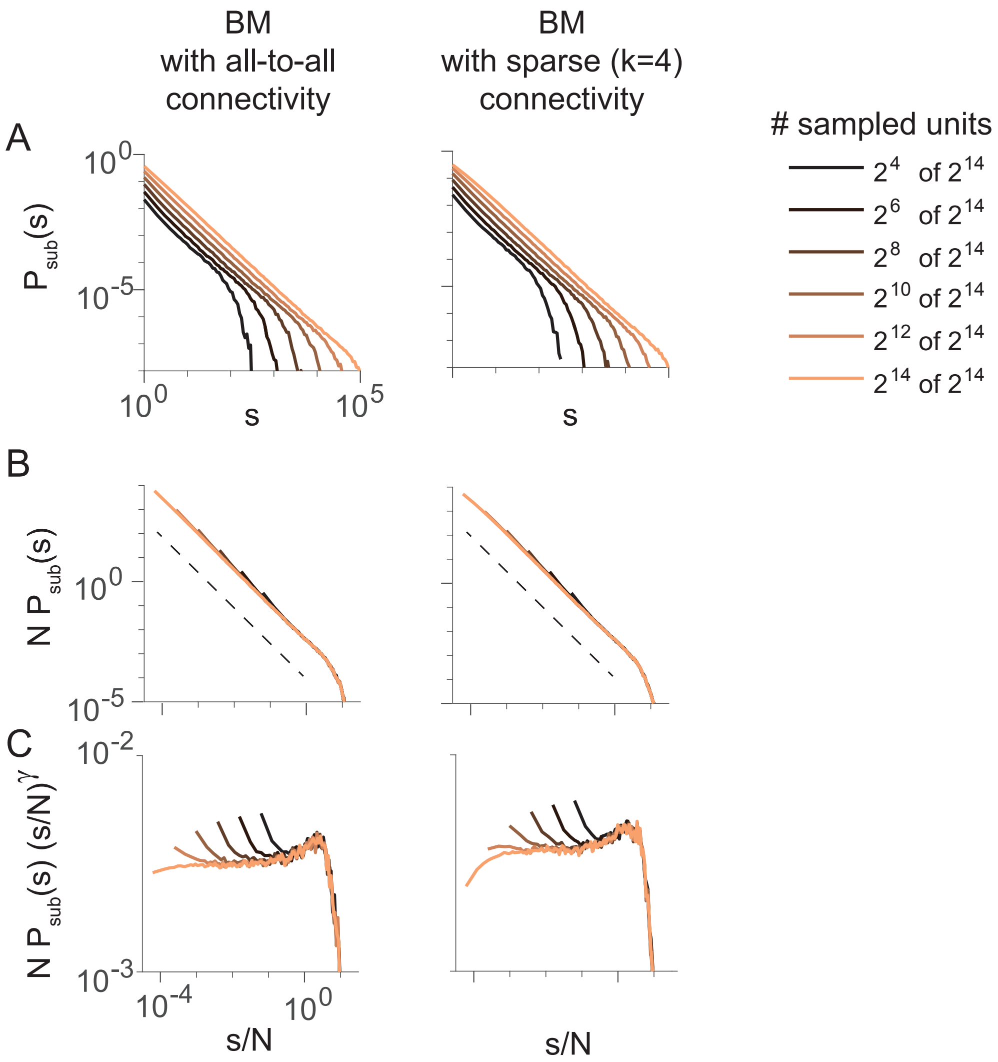

Supplementary Figure 6: Subsampling scaling in the fully and the sparsely connected branching model (BM), corresponding to Fig. 2 in the main text. Here avalanche size distributions $P_{\mathrm{sub}}(s)$ are shown for the BM with all-to-all connectivity (left), as in the main text, and for the BM with sparse, annealed connectivity with degree $k = 4$ (right). **A**: Both versions of the BM show very similar distributions, **B**: and a good collapse. **C**: In the flattened representation, minor differences between the two models become apparent, namely a lack of small avalanches s in the fully sampled, sparsely connected version of the BM. Both variants of the model have slope $\gamma = 1.5$ (dashed black line).

Supplementary Note 4. Detailed discussion of the experimental results

Supplementary Fig. 7 displays $P_{\mathrm{sub}}(s)$ for all recordings of developing neural cultures we evaluated (see Methods). As discussed in the main text (Fig. 4), with maturation the $P_{\mathrm{sub}}(s)$ approached power-law scaling, which for the fully sampled culture is expected to extend over almost six orders of magnitude. In addition to the power laws, about half of the mature cultures also showed a bump in $P_{\mathrm{sub}}(s)$ at very large sizes ($s \approx 5000$). These very large avalanches comprise only a tiny fraction of all avalanches ($\approx 0.02\%$). Such bumps are a priori not expected for critical systems. The collapse of the bumps itself is a manifestation of the activity spread during the large avalanches that hit the sampled set proportionally to the number of sampled units. In the following we discuss first whether the distributions with the bumps are expected to collapse under p-scaling, and then the potential origin of the bumps.

Regarding the questions whether the distributions observed here are expected to collapse, the answer is straight forward: The avalanches in the tail make only a tiny fraction of all observed avalanches (about 2 in 10,000), while the other 99.98% avalanches follow a power law for about 3 orders of magnitude. (It is the log-log scale together with the logarithmic binning that might make the bumps appear more prominent than they are.) With only 0.02% of avalanches not following a power law, a decent collapse is to be expected.

Regarding the origin of the bumps, there are a number of potential explanations, which we outline in the following. At first glance, the bumps are reminiscent of supercritical systems (hypothesis 3), however, they do not occur at N, the number of sampled units, as expected for supercritical systems. More likely, they may represent finite size effects (hypothesis 1), or alternatively transient switches to a bursty state (hypothesis 2). All three hypotheses are detailed in the following:

1. *Biological finite size effects in a critical system.*
   Assume the neural cultures were precisely at a critical point. Thus the distribution of the avalanche sizes would be a perfect power law without cutoff. However, in biological systems the avalanche size cannot go to infinity, because biological mechanisms (e.g. depletion of synaptic resources, shortage of Ca^{2+} or homeostatic mechanisms) limit their maximal size. All these avalanches that are larger than some s_{trans} (in our data $s_{\mathrm{trans}} \approx 3000$) are thus expected to be distributed around a characteristic, biologically determined size, which here is about $s \approx 5000$. The probability p_{bump} to observe an avalanche larger than some maximal size s_{trans} is given by the Hurwitz zeta function. Indeed, in agreement with this hypothesis, the number of the avalanches observed in the bump agrees with the probability p_{bump} for perfectly critical system. Thus the data support our hypothesis that the bump represents the collection of all avalanches that would, in an ideal system, be larger than 3000. (Note that all avalanche sizes s given here are the sizes observed under subsampling). Thus biological finite size effects are a probable origin for the bumps.

2. *Criticality alternates with a state that gives rise to large avalanches*
   The *in vitro* neural networks we analyzed could in principle alternate between different states. While in one state, which comprises about 99.98% of the avalanches, the system is critical, in the other state it displays unusually large

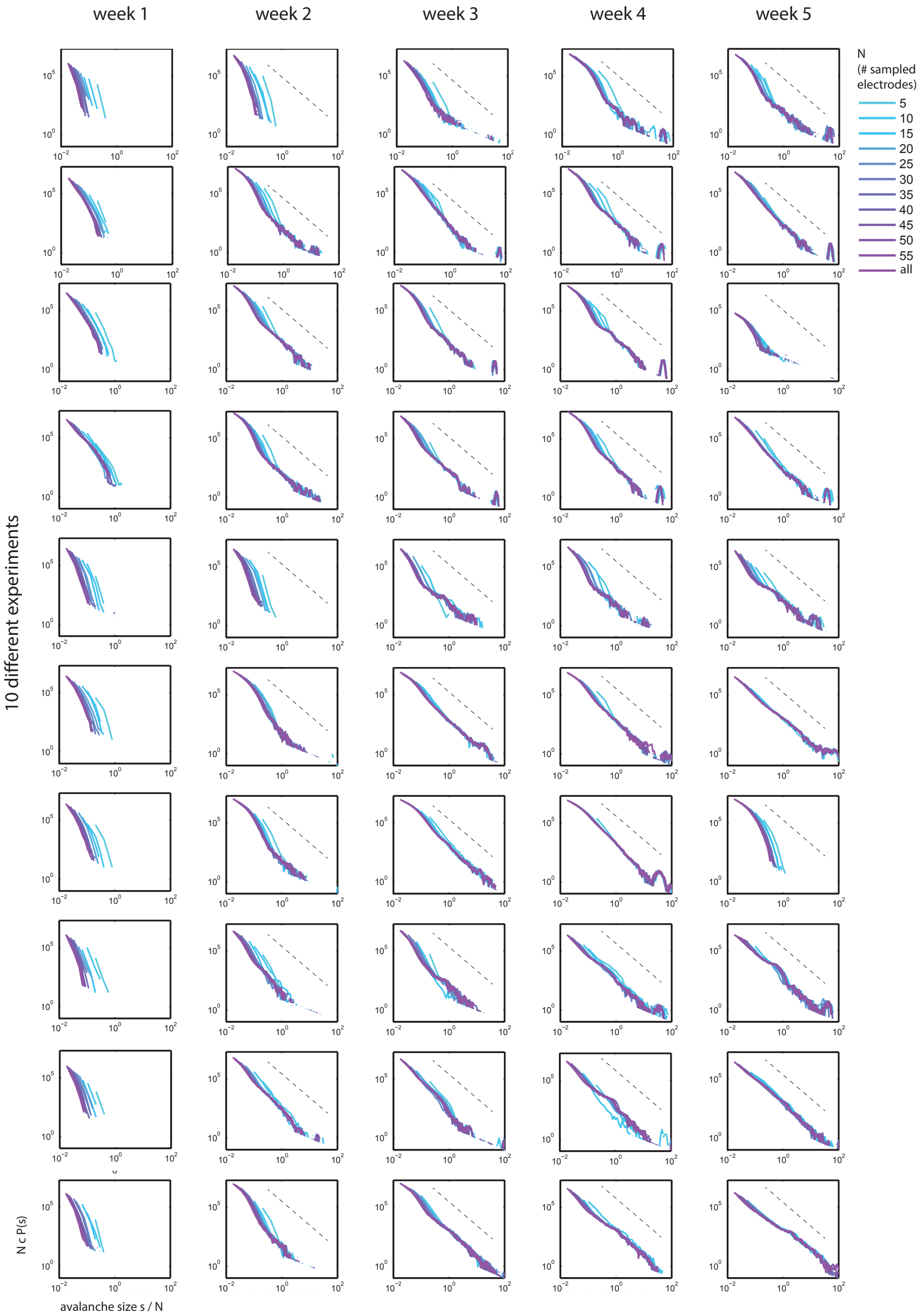

Supplementary Figure 7: Changes of the avalanche size distributions with development. This figure corresponds to Fig. 4 in the main text, but here shows distributions for all recordings we evaluated, and for all five recording weeks (typically day 7, 14, 21, 28, 34). For each experiment, the p-scaled avalanche size distributions $P_{\mathrm{sub}}(s)$ are displayed; c denotes the total number of avalanches observed in the respective recording, and the dashed line a slope of -2 for visual guidance.

avalanches that run multiple times over the entire system and give rise to population bursts, i.e. they manifest as the observed bumps. The precise fraction of "burst avalanche" can depend on the properties of each individual culture (some showing none at all), and it could be pure coincidence that the fraction of burst avalanches is in agreement with the fraction expected for the avalanche tail (see hypothesis 1).

3. *A novel form of slight supercriticality in a finite system.*
While it is straight forward to identify "*sub*criticality" (no avalanches covering the full system size, no power-law behavior of distributions, but a prominent exponential tail), it is much trickier to identify "supercriticality" in neural systems by pure observation, potentially because supercriticality in the thermodynamic limit implies a non-zero fraction of infinite avalanches, but in finite systems it depends on the type of system how these infinite avalanches manifest. For supercritical systems in neuroscience, the bump in $P_{\mathrm{sub}}(s)$ occurs typically at N, i.e. the system size or the number of sampled neurons [6, 10]. However, here in all experiments where the bump is observed, it is around 80 times N (i.e. $s \approx 5000$ from sampling up to 60 electrodes). Thus here the bumps do not indicate supercritical behavior resembling that of previous studies. However, it could indicate a novel form of supercriticality on a finite system.

How to distinguish between these potential causes of the bump appearance remains an open question for further experimental investigations (e.g. changing the network size; making the network on purpose supercritical). In the experiments evaluated here, the presence of the data collapse in the more mature networks predicts a power-law distribution for $P(s)$ of the full neural system that spans approximately 6 orders of magnitude. However, whether such power-laws scaling is sufficient to infer criticality, is still under debate.

Supplementary Note 5. Combining subsampling scaling and finite-size scaling

As demonstrated in section "Subsampling versus finite size scaling", there is a fundamental difference between subsampling scaling that deals with partial observations of a system, and finite-size scaling (FSS) that extrapolates from models of finite size to infinite size systems. Here we show how to combine both scaling ansätze to obtain a universal scaling.
The finite-size scaling ansatz for a critical system is formulated as:

$$P(s, M) = M^{-\beta} g\left(\frac{s}{M^{\nu}}\right) \Leftrightarrow M^{\beta} P(sM^{\nu}, M) = g(s), \tag{16}$$

where $g(s)$ is a scaling function. The formulation for the subsampling scaling in a system with M units is:

$$P_{\mathrm{sub}}(s, N; M) = N^{-1} g_{\mathrm{sub}}\left(\frac{s}{N}; M\right).$$

This can be re-written as:

$$NP_{\text{sub}}(sN, N; M) = g_{\text{sub}}(s; M) = MP_{\text{sub}}(sM, M; M). \quad (17)$$

Our goal is to combine the finite size scaling and the subsampling scaling relations (Eqs. 16 and 17) to factorize out the dependence of g_{sub} on M, and hence be able to collapse subsampled distributions from different system sizes M. To find the appropriate scaling, we need to identify the exponents δ and κ such that

$$NM^{\delta} P_{\text{sub}}(sNM^{\kappa}, N; M) = g(s).$$

To this end, recall that $P_{\text{sub}}(s, M; M) = P(s, M)$. In the following we first use subsampling scaling to express $P_{\text{sub}}(s, M; M)$ in terms of $P(s, M)$ using Eq. 17:

$$\begin{aligned} NM^{\delta} P_{\text{sub}}(sNM^{\kappa}, N; M) &= M^{1+\delta} P_{\text{sub}}(sM^{\kappa+1}, M; M) = \\ &M^{1+\delta} P(sM^{\kappa+1}, M) = M^{1+\delta-\beta} g(sM^{\kappa+1-\nu}). \end{aligned}$$

Thus the solutions for the exponents is given by $\delta = \beta - 1$ and $\kappa = \nu - 1$, and hence the general subsampling-finite-size scaling is given by:

$$NM^{\beta-1} P_{\text{sub}}(sNM^{\nu-1}, N; M) = g(s). \quad (18)$$

We tested this relation numerically for the case of the branching model (BM). For this model, FSS is given by $\beta = 1.5$, $\nu = 1$ and thus the subsampling-finite-size scaling is given by:

$$P(s) = NM^{0.5} P_{\text{sub}}(sN). \quad (19)$$

Indeed, with this scaling we obtained as expected a good collapse for combining different sampling sizes N and system sizes M (Supplementary Fig. 6).

Supplementary Note 6. Numerical estimation of optimal scaling

Throughout the manuscript we used an analytical approach to determine the optimal scaling for the subsampled distributions. In this section we confirm that our analytical results coincide with a direct numerical estimation of the scaling constants a, b.

To achieve an optimal scaling collapse, we numerically estimated the parameters a and b that minimize the distance $d(a, b)$ between the rescaled distributions $P_{\text{sub}}(s; a, b, N) = N^a P_{\text{sub}}(N^b s)$ under subsampling, and the rescaled distribution under full sampling, $P(s; a, b, N = M)$. In more detail, we first estimated for each $N \in [8, 16, 32, \ldots, M]$ the distance $d(a, b; N)$ as follows:

$$d(a, b, N) = \langle |\ln(P_{\text{sub}}(s; a, b, N)) - \ln(P(s'; a, b, M))| \rangle_s . \quad (20)$$

The mean $\langle \cdot \rangle_s$ was taken over all $s \in [1, 10 \cdot N]$, and s' are the support points in $P(s'; a, b, M)$ corresponding to those in $P_{\text{sub}}(s; a, b, N)$, i.e. $s'/M^b = s/N^b$ is fulfilled. As s' may take non-integer values, the values $P_{\text{sub}}(s'; a, b, N)$ are obtained, if necessary, by linear interpolation between the nearest integers. Then the weighted

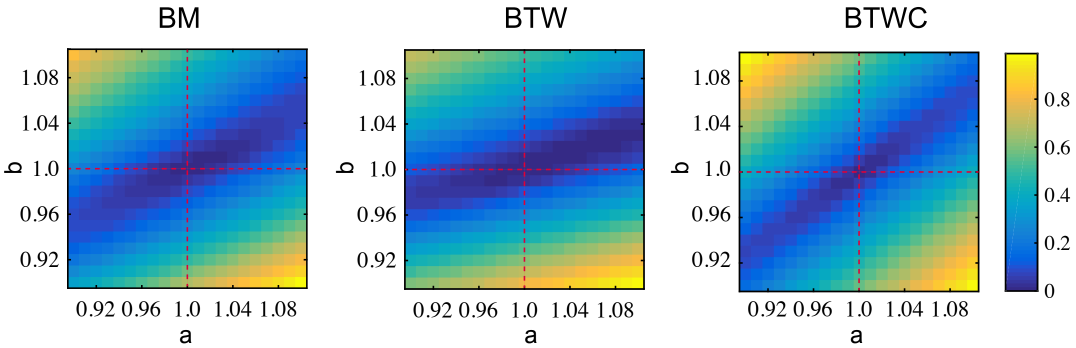

Supplementary Figure 8: Numerical estimation of scaling parameters a, b. Color code represent deviation from the perfect collapse $d(a, b)$, yellow – large deviation, dark blue – close to perfect collapse. Red dashed lines denote the analytical prediction. Left: Branching model (BM), middle: Bak-Tang-Wiesenfeld model (BTW), right: BTW with circular boundaries (BTWC). For presentation purposes $d(a, b)$ was shifted and scaled to the interval between zero (for the minimal value) and unity. This procedure does not change the location of the minima.

average over all $d(a, b, N)$ is taken to obtain $d(a, b) = \langle d(a, b, N) \rangle_N$. The parameter combination (a^*, b^*) that minimizes $d(a, b)$ provides numerically the optimal collapse. We scanned a and b in steps of 0.01 and found for both, the BM and the BTWC an optimal collapse at $a^* = b^* = 1$, as predicted analytically (Supplementary Fig. 8). For the BTW, the optimal collapse was at $a^* = 1.01, b^* = 1.02$, but the value of $d(a, b)$ in the point analytically obtained $a = b = 1$ deviated only by 3% from the absolute minimum (Supplementary Fig. 8). Thus overall, our numerical results match very well the theoretical prediction.

Supplementary References

[1] Stumpf, M. P. H., Wiuf, C. & May, R. Subnets of scale-free networks are not scale-free: sampling properties of networks. *PNAS* **102**, 4221–4224 (2005).

[2] Rudin, W. *Real and complex analysis.* Tata McGraw-Hill Education, (1987).

[3] Harris, T. E. *The theory of branching processes.* Springer, (Berlin, 1963).

[4] Eurich, C. W., Herrmann, M. & Ernst, U. Finite-size effects of avalanche dynamics. *Phys. Rev. E* **66**, 066137–1–15 (2002).

[5] Levina, A. *A mathematical approach to self-organized criticality in neural networks.* Niedersächsische Staats-und Universitätsbibliothek Göttingen, (2008).

[6] Beggs, J. & Plenz, D. Neuronal avalanches in neocortical circuits. *J. Neurosci* **23**, 11167–11177 (2003).

[7] Ribeiro, T. L., Ribeiro, S., Belchior, H., Caixeta, F. & Copelli, M. Undersampled Critical Branching Processes on Small-World and Random Networks Fail to Reproduce the Statistics of Spike Avalanches. *PloS one* **9**, e94992 (2014).

[8] Priesemann, V., Munk, M. & Wibral, M. Subsampling effects in neuronal avalanche distributions recorded in vivo. *BMC neuroscience* **10**, 40 (2009).

[9] Priesemann, V. *et al.* Spike avalanches in vivo suggest a driven, slightly subcritical brain state. *Frontiers in Systems Neuroscience* **8**, 108 (2014).

[10] Levina, A., Herrmann, J. M. & Geisel, T. Dynamical synapses causing self-organized criticality in neural networks. *Nature Physics* **3**, 857–860 (2007).
